# A SARS-CoV-2 Surveillance System in Sub-Saharan Africa: Modeling Study for Persistence and Transmission to Inform Policy

**DOI:** 10.2196/24248

**Published:** 2020-11-19

**Authors:** Lori Ann Post, Salem T Argaw, Cameron Jones, Charles B Moss, Danielle Resnick, Lauren Nadya Singh, Robert Leo Murphy, Chad J Achenbach, Janine White, Tariq Ziad Issa, Michael J Boctor, James Francis Oehmke

**Affiliations:** 1 Buehler Center for Health Policy & Economics and Departments of Emergency Medicine Feinberg School of Medicine Northwestern University Chicago, IL United States; 2 Feinberg School of Medicine Northwestern University Chicago, IL United States; 3 Division of Infectious Disease Feinberg School of Medicine Northwestern University Chicago, IL United States; 4 Institute of Food and Agricultural Sciences University of Florida Gainesville, FL United States; 5 International Food Policy Research Institute Washington, DC United States; 6 Institute for Global Health Northwestern University Chicago, IL United States

**Keywords:** global COVID-19 surveillance, African public health surveillance, sub-Saharan African COVID-19, African surveillance metrics, dynamic panel data, generalized method of the moments, African econometrics, African SARS-CoV-2, African COVID-19 surveillance system, African COVID-19 transmission speed, African COVID-19 transmission acceleration, COVID-19 transmission deceleration, COVID-19 transmission jerk, COVID-19 7-day persistence, Sao Tome and Principe, Senegal, Seychelles, Sierra Leone, Somalia, South Africa, South Sudan, Sudan, Suriname, Swaziland, Tanzania, Togo, Uganda, Zambia, Zimbabwe, Gambia, Ghana, Guinea, Guinea-Bissau, Kenya, Lesotho, Liberia, Madagascar, Malawi, Mali, Mauritania, Mauritius, Mozambique, Namibia, Niger, Nigeria, Rwanda, Angola, Benin, Botswana, Burkina Faso, Burundi, Cameroon, Central African Republic, Chad, Comoros, Congo, Cote d'Ivoire, Democratic Republic of Congo, Equatorial Guinea, Eritrea, Ethiopia, Gabon

## Abstract

**Background:**

Since the novel coronavirus emerged in late 2019, the scientific and public health community around the world have sought to better understand, surveil, treat, and prevent the disease, COVID-19. In sub-Saharan Africa (SSA), many countries responded aggressively and decisively with lockdown measures and border closures. Such actions may have helped prevent large outbreaks throughout much of the region, though there is substantial variation in caseloads and mortality between nations. Additionally, the health system infrastructure remains a concern throughout much of SSA, and the lockdown measures threaten to increase poverty and food insecurity for the subcontinent’s poorest residents. The lack of sufficient testing, asymptomatic infections, and poor reporting practices in many countries limit our understanding of the virus’s impact, creating a need for better and more accurate surveillance metrics that account for underreporting and data contamination.

**Objective:**

The goal of this study is to improve infectious disease surveillance by complementing standardized metrics with new and decomposable surveillance metrics of COVID-19 that overcome data limitations and contamination inherent in public health surveillance systems. In addition to prevalence of observed daily and cumulative testing, testing positivity rates, morbidity, and mortality, we derived COVID-19 transmission in terms of speed, acceleration or deceleration, change in acceleration or deceleration (jerk), and 7-day transmission rate persistence, which explains where and how rapidly COVID-19 is transmitting and quantifies shifts in the rate of acceleration or deceleration to inform policies to mitigate and prevent COVID-19 and food insecurity in SSA.

**Methods:**

We extracted 60 days of COVID-19 data from public health registries and employed an empirical difference equation to measure daily case numbers in 47 sub-Saharan countries as a function of the prior number of cases, the level of testing, and weekly shift variables based on a dynamic panel model that was estimated using the generalized method of moments approach by implementing the Arellano-Bond estimator in R.

**Results:**

Kenya, Ghana, Nigeria, Ethiopia, and South Africa have the most observed cases of COVID-19, and the Seychelles, Eritrea, Mauritius, Comoros, and Burundi have the fewest. In contrast, the *speed*, *acceleration*, *jerk*, *and 7-day persistence* indicate rates of COVID-19 transmissions differ from observed cases. In September 2020, Cape Verde, Namibia, Eswatini, and South Africa had the highest speed of COVID-19 transmissions at 13.1, 7.1, 3.6, and 3 infections per 100,0000, respectively; Zimbabwe had an acceleration rate of transmission, while Zambia had the largest rate of deceleration this week compared to last week, referred to as a *jerk*. Finally, the 7-day persistence rate indicates the number of cases on September 15, 2020, which are a function of new infections from September 8, 2020, decreased in South Africa from 216.7 to 173.2 and Ethiopia from 136.7 to 106.3 per 100,000. The statistical approach was validated based on the regression results; they determined recent changes in the pattern of infection, and during the weeks of September 1-8 and September 9-15, there were substantial country differences in the evolution of the SSA pandemic. This change represents a decrease in the transmission model R value for that week and is consistent with a de-escalation in the pandemic for the sub-Saharan African continent in general.

**Conclusions:**

Standard surveillance metrics such as daily observed new COVID-19 cases or deaths are necessary but insufficient to mitigate and prevent COVID-19 transmission. Public health leaders also need to know where COVID-19 transmission rates are accelerating or decelerating, whether those rates increase or decrease over short time frames because the pandemic can quickly escalate, and how many cases today are a function of new infections 7 days ago. Even though SSA is home to some of the poorest countries in the world, development and population size are not necessarily predictive of COVID-19 transmission, meaning higher income countries like the United States can learn from African countries on how best to implement mitigation and prevention efforts.

**International Registered Report Identifier (IRRID):**

RR2-10.2196/21955

## Introduction

### Background

In December 2019, a novel illness leading to severe pneumonia and acute respiratory disease was observed in Wuhan, China. The etiologic pathogen was identified as a novel coronavirus, SARS-CoV-2, and the associated respiratory illness was named COVID-19 [1]. As cases began to spread from China, the outbreak was recognized as a global health emergency by the World Health Organization (WHO). Within weeks, the economic significance, clinical manifestations, and the burden on local health systems began to be felt universally. To date (September 27, 2020), over 33 million cases have been reported worldwide, with 128.1 deaths per 1 million [2]. Africa as a whole has reported 1.45 million cases, while the United States has reported over 7.2 million cases, India over 5.9 million, and Brazil over 4.7 million [2].

Using commonalities in geography, development, economies, and climate, the World Bank encompasses 48 countries within the sub-Saharan Africa (SSA) region [3]. The countries of SSA today (September 27, 2020) report 1,131,385 total COVID-19 cases and 26,285 COVID-19–related deaths [2]. SSA comprises immense diversity among the 750 million people that reside in the region. Africa has more than 2000 separate and distinct languages, as well as numerous cultural and ethnic groups with their own history, traditions, and religion [4]. These countries also vary in governance, political stability and unrest, infrastructure, medical resources, and public health [5]. Given their proximity and heterogeneity, SSA countries face additional challenges implementing unified policies to combat the pandemic.

**Figure 1:**
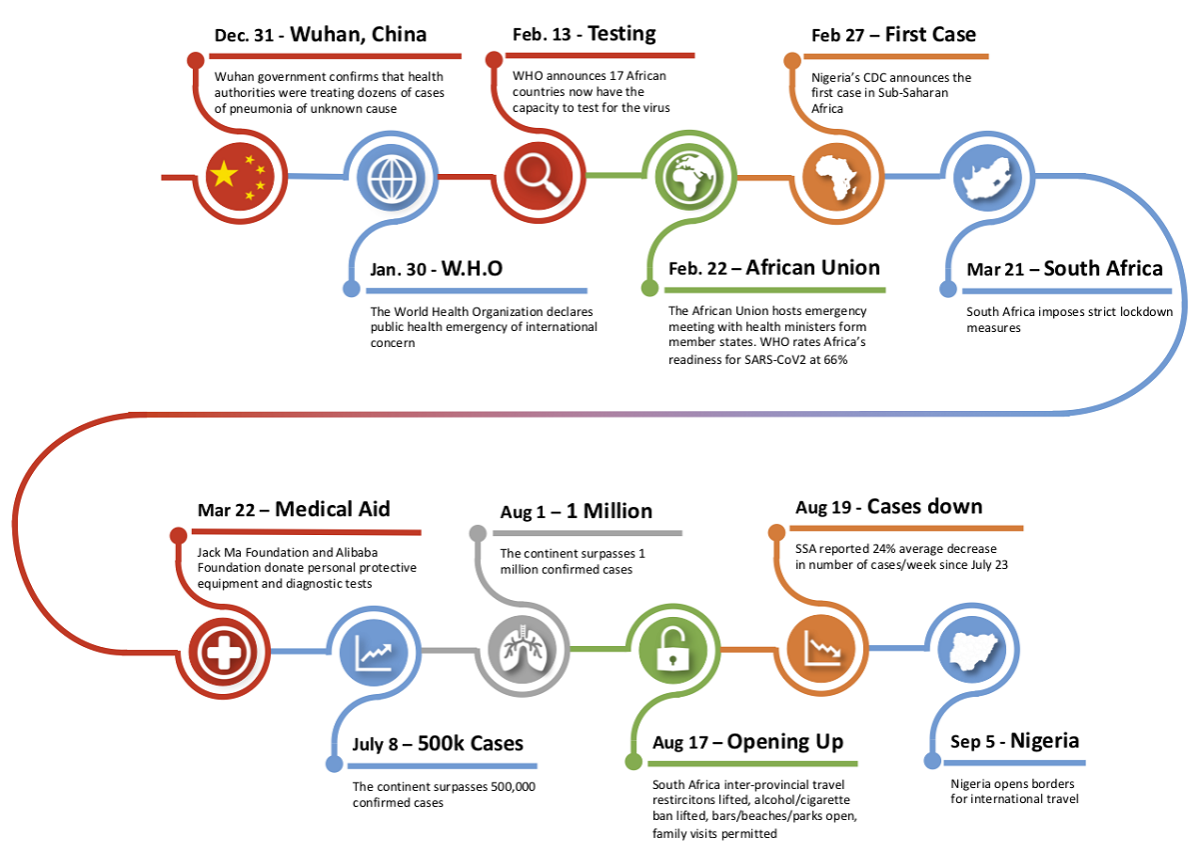
Sub-Saharan Africa COVID timeline.

The first reported case of COVID-19 in SSA occurred in Nigeria in February 2020 (Figure 1) [6]. The case was an Italian citizen who returned to Lagos, Nigeria for work after visiting Italy. The Nigerian Minister of Health published health precautions following the positive test result encouraging all Nigerians to take safety measures such as hand washing and social distancing seriously [6]. Since then, Nigeria has reported over 55,000 cases with 5 deaths per 1 million [2]. South Africa has thus far reported the greatest number of cases in SSA at nearly 642,000 cases, with countries like Seychelles, Eritrea, and Mauritius reporting the least for the region at less than 500 cases [2].

SSA has vast experience in tracking and treating infectious outbreaks, with 45% of the continent facing at least one epidemic per year [7]. Countries have been able to leverage this experience to effectively ramp up diagnostic testing for COVID-19 and reinforce previously communicated public health practices such as hand hygiene and social distancing [8]. In Nigeria, tuberculosis (TB) and HIV testing technologies have been converted for COVID-19 use, and in Ethiopia, Abbott reconfigured instruments to increase COVID-19 testing capacity to 7600 tests per day [9]. Through such measures, African countries increased diagnostic capacity from 2 to 43 countries in 3 months and are currently working to further increase subnational capacity [9].

Urban areas must also mitigate the transmission of the virus within a context of high population density and mobility. The rapid urbanization of SSA cities has resulted in nearly 60% of urban dwellers (336 million people) living in informal settlements and slums [10]. SSA must cope with all of these challenges along with the burden of its inconsistent infrastructure and policy systems (ie, inequalities in access to safe water and sanitation, drought impacts, access to health care facilities), high population to medical provider ratio, and fragile economies vulnerable to lockdown costs in facing this pandemic [10].

### Demographic and Geographic Profile

**Figure 2:**
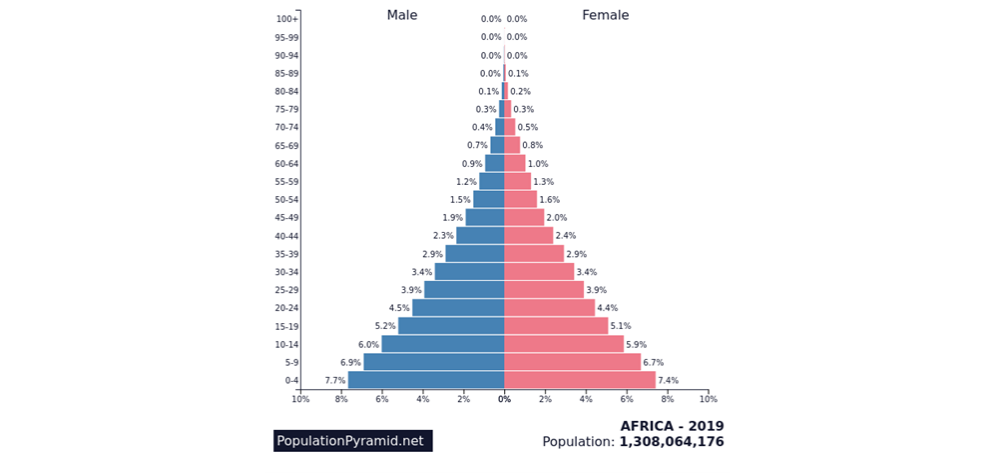
Population pyramid for sub-Saharan Africa.

SSA has high fertility combined with a decrease in infant and child mortality, which results in a short and wide base in the population pyramid (see Figure 2) [11]. Compared to high income and developed countries, SSA is a youthful population. SSA is home to 37 of the world’s youngest countries with nearly 60% of the population younger than 25 years and more than 90% of its population younger than 50 years [12] (see Figure 2). Although younger people are less likely than older adults to develop severe outcomes of COVID-19, it is unclear if SSA’s youthful population benefits from age-related protective factors due to the health effects of food insecurities and the high communicable disease rate (eg, HIV/AIDS, TB, and malaria) [12]. Some research suggests that the young average age of the population and the prominence of rural dwellings may limit the overall spread and severity of the pandemic, perhaps outweighing the negative impact incurred by the high communicable disease burden [5]. The pervasiveness of rural dwellers, however, also increases the likelihood of staggered outbreaks, requiring vigilance in policy making [5]. Children are just as likely as adults to become infected with SARS-CoV-2 but are less likely to be symptomatic or develop severe symptoms [13-15]. Thus, when comparing SSA to Europe, Asia, and North America, SSA has a significantly younger population structure [16].

Population density is another COVID-19 demographic risk factor. The closer people live to each other and the more they interact, the greater the likelihood of transmitting the novel coronavirus [17]. Even though the African continent has experienced significant growth due to the high fertility rates and lower mortality rates, Africa still has several sparsely populated areas. In 2017, the average population density was 16.6 persons per square kilometer. Today, the average population density of the whole world is 51 persons per square kilometer, and 23 African countries rank below the global average. Moreover, most of the African countries with the highest population density are smaller countries such as Rwanda, Comoros, Burundi, and Mauritius [18].

Out of the more than 1 billion people that live in SSA, there are 2,213,327 more females than males. Worldwide, males generally have experienced worse outcomes from COVID-19 than females [19-21].

### Policies and Governance

As early as January 2020, countries such as Côte d’Ivoire (Ivory Coast) began implementing symptom checks at airports and travel restrictions from China [22]. In February, the Africa Centres for Disease Control and Prevention (Africa CDC) organized the *Africa Joint Continental Strategy: COVID-19 Outbreak among the African Union*. Member states created an international task force aimed at preventing mortality and morbidity while abating social disturbance and economic penalties [23]. This task force has since recommended evidence-based policies, worked to procure diagnostic tools, organized workshops for containment and mitigation strategies, and provided training and advice for public health systems [8,22]. By the end of May, most African countries had banned flights from Asia and Europe as well as implemented quarantine periods for other travelers and placed restrictions on school and other public gatherings, in addition to executing lockdowns and curfews [22].

Although most SSA countries acted promptly and decisively, others have been more inert. Tanzania’s President John Magufuli, for example, declared diagnostic tests defective in May after samples from goats and sheep returned positive [24]. Soon after, the country abandoned reporting new cases (last reported number at 509) and in June declared itself virus-free [25]. Subsequently, neighboring countries (ie, Kenya, Uganda, and Zambia) tightened their borders in an attempt to control spread, especially with truck driver mobility [24]. In addition, Uganda and Kenya set curfews and partial or full lockdowns alongside designated isolation facilities [24,26]. Similarly, Zambia set aside two medical facilities in the capital for the purpose of quarantining, and the Democratic Republic of the Congo closed provincial borders, churches, and educational facilities in addition to implementing curfews [26,27]. This was in contrast to Tanzania’s open churches and mosques as well as public transportation despite an initial ban on public gatherings [28,29]. Still, in other countries the spread of false or unconfirmed information by politicians and public figures about coronavirus “cures” such as the herbal tonic promoted by President Andry Rajoelina of Madagascar caused confusion and some preventable deaths [30].

Another major concern has been the use of oppressive actions by law enforcement to contain outbreaks. There have been reports in Kenya of civilian deaths by security forces in the process of enforcing curfews [31]. Uganda similarly reported attacks on lesbian, gay, bisexual, transgender, and queer individuals in a shelter for failure to social distance [31]. Uganda, Nigeria, and the Democratic Republic of Congo have seen the largest increase in violence targeting civilians since the pandemic onset compared to the immediate prepandemic period [32]. At the expense of civil liberties, South Africa and Mozambique set restrictions on the spread of misinformation around the outbreak. Ethiopia postponed elections in an effort to minimize large gatherings, and Ghana banned political rallies in advance of its December 2020 elections [12,33]. Achieving the delicate balance between protecting human rights and limiting misinformation while effectively containing outbreaks in countries with a history of oppressive governments and political unrest will be critical to regaining stability in the postcoronavirus era.

### Health Systems

Public health systems must confront provider shortages, limited health facilities, and medical supply shortages in meeting community needs [34]. Rural residents especially have to travel longer distances and have worse access to health care due to these shortages [34]. On average, African countries have 1.8 hospital beds per 1000 people. South Africa has 2.8, Botswana 1.8, Nigeria 0.5, Ethiopia 0.3, and Ghana 0.9 [35]. Uganda has 55 intensive care unit beds for 40 million people with added limitations due to a dearth in critical care doctors and anesthetists [36]. South Africa has 0.9 physicians per 1000 people, Botswana 0.5, Nigeria 0.4, and Ethiopia and Ghana 0.1 [35]. In comparison, the United States has 2.6, Italy has 4.0, and China has 2.0 physicians per 1000 people [37]. Armed conflicts in South Sudan after nearly a decade of conflict have weakened the health system with damage to infrastructure, depletion of workforce, and reallocation of funds toward national security [38]. In Burkina Faso, displacement of people and increased migration across its borders has similarly complicated the health system’s ability to meet health needs [38].

Furthermore, many SSA countries are not able to screen for COVID-19 using constitutional symptoms like fevers, as they face high concurrent infection rates [5,36]. The current pandemic necessitates redirection of resources from other public health initiatives to COVID-19, allowing countries to meet imminent needs at the expense of others. Countries like Nigeria have been able to effectively combat COVID-19 by transitioning infrastructure (ie, laboratory services, surveillance, and human resources) and converting diagnostic tests previously meant for poliovirus, as they had done during the Ebola outbreak [8,38,39]. Although this is innovative and highly effective, it does come with consequences: the Ebola outbreak was later associated with a decrease in hospital births and an increase in maternal mortality rates [10]. Recent news has similarly revealed major concerns around maternal mortality in light of COVID-19 precautions [40].

There is a global shortage of personal protective equipment (ie, face masks, plastic and rubber gloves, and surgical garments), diagnostic tools, and ventilators, which is more pronounced in SSA due to dependencies on imported medical and pharmaceutical products [38]. African countries as a whole import a total of US $748 million in protective garments and US $9291 million in disinfectants and sterilization products annually [10]. Countries worldwide have placed limitations on the export of supplies they deem necessary for tackling COVID-19 internally. These include protective masks, ventilators, medicines, disinfectants and sterilization products, intubation products, and paper bedsheets among others [10]. Although this adds to the concerns for the region, the WHO, Jack Ma Foundation, and Alibaba Foundation in Ethiopia, among many others, have continued to donate medical equipment and diagnostic kits to mitigate burdens on African Union states [26,41].

### Economics

The World Bank estimates 70-100 million people worldwide may be pushed below the poverty level as a result of the COVID-19 pandemic, and anywhere from 26-39 million of them reside in SSA, which is now projected to experience its first recession in 25 years [42]. The economic effects of the pandemic are likely to impact younger people in the sub-Saharan workforce who provide physical labor [43]. In South Africa, the government has imposed several waves of lockdowns and closures, and last month was forced to reimpose a curfew when cases rebounded. The South African Chamber of Commerce and Industry has projected unemployment as high as 50% in coming months, and the economy is projected to shrink 7% this year [44]. The novel coronavirus has also delayed the implementation of the African Continental Free Trade Area implementation and instead has led to a series of border closures throughout the region [45]. The economic policy responses to COVID-19 have varied significantly between nations and have involved a number of different tactics: interest rate cuts, fee waivers, and direct relief. A majority of sub-Saharan national governments are now offering some level of income support for lost wages as a result of the pandemic, though only Gabon and Benin are providing more than 50% of lost salary. In addition, a number of the region’s most populous nations including Nigeria, Ethiopia, the Democratic Republic of Congo, Tanzania, and South Africa, are currently offering no wage relief [46].

### Food Security

The impact of COVID-19 on food security is both direct and indirect: agricultural production itself is projected to contract from 2% to 7% alongside decreased food imports resulting from increased costs associated with border closures and disrupted supply chains. An analysis of Ghana’s 3-week lockdown in March found the agrifood system gross domestic policy, which incorporates primary agriculture as well as food trade and food service industries, declined 19.8%, even though primary agricultural activities were excluded from lockdown measures [47]. Nigeria also saw a similar drop in agrifood gross domestic product during its own lockdown [48]. The primary reason for a projected increase in acute hunger is simply falling incomes resulting from the economic fallout previously described [42]. Those near or below the poverty line spend a larger share of their income on food, and any decline in incomes is likely to directly impact their access to food [49]. Food prices have begun to rise, which may leave struggling residents with fewer calories and a need to substitute with cheaper, less nutritious options. Indeed, a study conducted in Addis Ababa, Ethiopia in May found that one-fourth of all households reported reducing their household food expenditures over the previous month, and the biggest decline in spending was on fresh produce [50]. Eastern Africa countries including Ethiopia, Kenya, and Tanzania have faced large-scale crop devastation due to locusts and flooding. Budget shortfalls have limited food distribution by the World Food Programme and the United Nations Commissioner for Refugees in the region, affecting the 3.2 million refugees currently residing in Ethiopia, Kenya, Sudan, South Sudan, and Tanzania. A recent survey of South Sudanese refugees found 80% were resorting to rationing or skipping meals, and refugee camps in Rwanda have reported a 27% increase in food prices. So-called transitional food supply chains, which feature many links and often end at small enterprises and wet markets, are the predominant pattern in much of SSA and will likely struggle to comply with stringent government-imposed regulation [51].

### Sociocultural

There are a number of social and cultural factors that affect both the impact of COVID-19 on SSA and the response to the pandemic. Infrastructure challenges pose a barrier on a number of fronts: a lack of piped water limits hand hygiene, uneven personal transportation options impede access to resources during lockdowns, and overcrowded housing in urban and semiurban areas make social distancing impossible [7]. There is also a fear that lockdowns and closures may exacerbate certain structural inequalities. School closures threaten long-term educational attainment, and socially distant learning is simply not an option in locales with limited or nonexistent internet access. According to data from the International Telecommunications Union, Africa has the largest share of the world’s offline population, with an overall internet penetration rate of around 28% [52]. Gender-based violence is also expected to increase with prolonged lockdowns, and the pandemic threatens to reverse hard-fought economic and social advances in gender equality in recent decades [40,53,54]. A final psychosocial factor playing out in real time is the impact of recent previous infectious disease outbreaks such as Ebola and HIV. Although this recent experience has in some cases led to more aggressive policy responses than in other global regions, many of the public health response challenges for Ebola, as well as the mistrust of health care workers and stigmatization of victims and survivors, have persisted amid the COVID-19 response [55].

### Long-Term Outcomes of COVID-19

The legacy of the SARS-CoV-2 pandemic in SSA will depend on the morbidity and mortality of the pandemic but also the response. Currently, South Africa accounts for over half of reported cases in SSA, and although limited testing and surveillance may put those numbers into question, it appears that much of the region has yet to experience a large outbreak [56,57]. As previously outlined, many of the region’s countries acted decisively and swiftly to impose lockdown measures even as case numbers were still relatively low. The region now, however, faces the ongoing dilemma of confronting worsening poverty and food insecurity while also maintaining some public health measures to limit a renewed wave of infection. The lack of health care infrastructure remains a significant concern, and the informal and labor-intensive character of the region’s economy makes lockdowns, that wealthier nations implement, unsustainable. Public health experts fear the upshot of the pandemic may be a resurgence in malaria, TB, and other deadly diseases [58]; however, there remains hope that the response to COVID-19 and the upheaval resulting from the virus may facilitate creative and inclusive initiatives that not only limit the effects of the virus but also contribute to a sustainable recovery and growth [12].

Scientists continue to praise Africa’s quick response to closing international borders and enforcing strict quarantine and social distancing measures in many countries even before any deaths were reported [59,60]. Since August 1, 2020, nations in SSA have seen an attenuation in many of the public health and social measures responsible for the incredible early success in combatting the virus [61]. Countries have begun to lift their lockdowns and ease back into a prepandemic form of life. Earlier in September, Zimbabwe recorded no COVID-19–related deaths over an entire week [61]; moreover, many residents have stopped wearing masks, and the government eased its restrictions. As Nigeria similarly began to see a decline in cases, they opened their borders for international travel on September 5, 2020 [62].

With reported cases falling in South Africa, the government reduced its national alert level from level 3 to level 2 on August 17, 2020, lifting travel restrictions between provinces; allowing bars, taverns, gyms, and beaches to open; and ending the ban of alcohol and cigarette sales [63,64]. After continued reduced disease spread, the government further reduced its national alert level to level 1 on September 20, 2020, increasing the capacity allowed within event venues [65]. As these restrictions continue to be eased across the continent, their effectiveness and impact on the spread of SARS-CoV-2 are still to be determined.

### Objective

Several organizations have developed surveillance and tracking tools for SARS-CoV-2 cases in Africa. These include the WHO Regional Office of Africa, the Africa CDC, the Worldometer, the Tony Blair Institute for Global Change, Johns Hopkins University, Statista, and the New York Times [2,7,22,66-69]. Although these data repositories offer good proxies for COVID-19 morbidity and mortality, they have undercounts, errors, and reporting biases. To that end, the objective of our research is to derive speed, acceleration and deceleration, jerk, and 7-day persistence to measure where and how rapidly COVID-19 is transmitting. We will rely on dynamic panel modeling and generalized method of the moments that correct for data limitations. Our study will measure the underlying causal effect from the previous week that persists through the current week with a 7-day persistence rate that explains new infections in the current week as a direct result of infections from the prior week. In SSA, for example, a 7-day persistence can occur following protest against postponed elections as was seen in Guinea this March. In summary, we will measure negative and positive shifts in the transmission of SARS-CoV-2 or the acceleration and deceleration rates that do not have sampling bias.

## Methods

The Foundation for Innovative New Diagnostics [70] compiles data from multiple sources across individual websites, statistical reports, and press releases; data for the most recent 8 weeks were accessed from the GitHub repository [71]. This resulted in a panel of 47 countries with 60 days in each panel (n=2820). An empirical difference equation was specified in which the number of positive cases in each state at each day is a function of the prior number of cases, the level of testing, and weekly shift variables that measure whether the contagion was growing faster, slower, or stayed the same than the previous weeks. This resulted in a dynamic panel model that was estimated using the generalized method of moments approach by implementing the Arellano-Bond estimator in R (the R Foundation for Statistical Computing) [72,73].

Arellano-Bond estimation of difference equations has several statistical advantages: it allows for statistical examination of the model’s predictive ability and the validity of the model specification, it corrects for autocorrelation and heteroscedasticity, it has good properties for data with a small number of time periods and large number of countries, and it corrects for omitted variables issues and provides a statistical test of correction validity. With these advantages, the method is applicable to ascertaining and statistically validating changes in the evolution of the pandemic within a period of a week or less, such as changes in the reproduction rate [74].

## Results

### Country Regression Results

We group 47 countries into the global region of SSA and present the regression results. The weekly surveillance products will be based on these regressions.

For SSA, the regression Wald statistic showed that the model was statistically significant (*χ*^2^_4_=289, *P*<.001), and the Sargan test failed to reject the validity of the overidentifying restrictions *χ*^2^_778_=47, *P*>.99; Table 1).

**Table 1:** Arellano-Bond dynamic panel data modeling of the number of daily infections reported by country, September 1-15, 2020.

Variable	Coefficient	*P* value
L1Pos^a^	0.052	.20
L7Pos^b^	0.069	.11
Cumulative Tests	0.013	<.001
Shift parameter week of September 8	–0.359	<.001
Shift parameter week of September 15	–1.05	<.001
Weekend	–0.131	.19

^a^L1Pos: 1-day persistence. The number of new infections today that are statistically related to those new infections 1 day ago per 100,000 population. Infections 1 day ago has an echo forward effect in COVID-19 transmissions that we call 1-day persistence rate.

^b^L7Pos: 7-day persistence. The number of new infections today that are statistically related to those new infections 7 days ago per 100,000 population. Infections 7 days ago has an echo forward effect in COVID-19 transmissions that we call 7-day persistence rate.

The coefficient on the first persistence of the dependent variable was not statistically significant, nor were the shift parameters for the weeks of September 8 and 15, 2020, for this coefficient. The coefficient on the seventh persistence was not statistically significant. This is consistent with the COVID-19 transmission rates. Overall, the rates are not changing significantly in SSA. The shift parameter for the weeks of September 8 and 15 were statistically significant. The cumulative number of tests administered was significant (coefficient 0.013, *P*=.001). The weekend variable was significant (coefficient –0.131, *P*=.19).

### Interpretation: SSA Regression Results

SSA overall appears to be fairly calm, with the only statistically significant effect being cumulative testing and the weekend effect. Both static surveillance metrics such as new cases of COVID-19 infections, cumulative cases, and deaths indicate a leveling or declining effect, but some countries appear to have accelerated growth in rates of COVID-19 transmissions.

### Surveillance Results

Surveillance results are presented in Tables 2-5. The four novel surveillance indicators are calculated as weekly averages to compare the transmission of COVID-19 from week to week (see Tables 2 and 3). These surveillance system data elements include average weekly number of daily positive tests per 100,000 population, referred to as *speed*; weekly average of day-to-day change in the number of positives per day per 100,000 population, referred to as *acceleration*; change in acceleration, referred to as *jerk,* which is the acceleration in the current week less the acceleration in the prior week (a sustained positive jerk is typically associated with explosive growth); and the *7-day persistence*, which is the number of new cases of COVID-19 reported on the current day per 100,000 population (ie, current speed) that are associated with new cases reported 7 days ago (ie, last week’s speed), measures how much an increase in speed in the previous week persists into the current week. Data are presented according to SSA countries. These novel indicators are standardized per 100,000 population to compare changes in rates of COVID-19 transmission between countries.

**Table 2:** Surveillance metrics for the week of September 1-8, 2020.

Country	*Speed*: daily positives per 100,000 (weekly average of new daily cases per 100,000), n	*Acceleration*: day-to-day change in the number of positives per day (weekly average per 100,000)	*Jerk*: week over week change in *acceleration* (per 100,000)	*7-day persistence effect* on *speed* (new cases per day per 100,000), n
Angola	0.14	–0.01	0.00	6.34
Benin	0.08	0.00	0.00	0.43
Botswana	2.49	–0.56	–1.33	2.30
Burkina Faso	0.07	0.00	0.00	0.45
Burundi	0.03	0.00	0.00	0.21
Cameroon	0.24	–0.01	–0.01	6.20
Cape Verde	11.17	–1.14	–1.04	5.71
Central African Republic	0.07	0.02	0.05	0.28
Chad	0.03	0.00	0.00	0.31
Comoros	0.49	0.00	0.00	0.14
Congo-Brazzaville (Republic of Congo)	2.42	0.00	–0.70	0.00
Congo-Kinshasa (Democratic Republic of Congo)	0.03	0.01	0.01	3.03
Cote d’Ivoire	0.37	0.02	0.03	7.69
Equatorial Guinea	0.21	–0.25	–0.39	0.55
Eritrea	0.09	0.04	0.05	0.06
Eswatini (Swaziland)	3.56	–0.26	–0.45	4.14
Ethiopia	0.95	0.00	0.00	136.70
Gabon	0.49	0.00	0.14	1.76
Gambia	1.50	0.07	0.07	4.88
Ghana	0.26	–0.01	–0.01	10.56
Guinea	0.41	–0.04	–0.02	4.99
Guinea-Bissau	0.30	0.00	0.00	0.80
Kenya	0.28	0.01	0.02	21.50
Lesotho	0.42	0.00	0.00	0.51
Liberia	0.02	0.00	–0.01	0.14
Madagascar	0.25	–0.01	–0.01	6.85
Malawi	0.04	0.00	0.02	2.18
Mali	0.08	0.01	–0.01	0.91
Mauritania	0.28	–0.09	–0.06	1.63
Mauritius	0.06	0.06	0.07	0.11
Mozambique	0.29	–0.02	–0.02	7.55
Namibia	7.08	–0.14	0.21	21.78
Niger	0.00	0.00	0.00	0.04
Nigeria	0.09	0.00	0.00	20.57
Rwanda	0.34	–0.06	–0.05	8.60
Sao Tome and Principe	0.13	0.00	0.00	0.06
Senegal	0.34	–0.01	0.01	8.52
Seychelles	0.15	0.00	–0.15	0.00
Sierra Leone	0.07	0.01	0.00	0.38
Somalia	0.06	0.01	0.01	0.50
South Africa	2.97	–0.03	0.24	216.68
South Sudan	0.03	0.01	0.02	0.28
Sudan	0.08	0.00	0.00	3.06
Togo	0.17	0.01	0.01	1.52
Tanzania	—^a^	—	—	—
Uganda	0.28	0.02	0.00	8.69
Zambia	0.46	–0.13	–0.12	15.58
Zimbabwe	0.81	0.03	–0.34	5.16

^a^Tanzania does not report COVID-19 cases.

**Table 3:** Surveillance metrics for the week of September 9-15, 2020.

Country	*Speed*: daily positives per 100,000 (weekly average of new daily cases per 100,000), n	*Acceleration*: day-to-day change in the number of positives per day (weekly average per 100,000)	*Jerk*: week over week change in *acceleration* (per 100,000)	*7-day persistence effect* on *speed* (new cases per day per 100,000), n
Angola	0.24	0.04	0.02	4.32
Benin	0.07	0.00	0.00	0.97
Botswana	2.09	0.00	–0.54	5.71
Burkina Faso	0.19	0.01	0.01	1.36
Burundi	0.01	0.00	0.00	0.30
Cameroon	0.23	–0.11	–0.14	6.24
Cape Verde	13.09	0.60	0.65	6.11
Central African Republic	0.11	–0.02	–0.09	0.34
Chad	0.04	0.00	0.00	0.40
Comoros	0.18	0.10	0.08	0.41
Congo-Brazzaville (Republic of Congo)	0.11	0.00	0.68	12.97
Congo-Kinshasa (Democratic Republic of Congo)	0.02	–0.01	0.00	2.67
Cote d’Ivoire	0.18	–0.02	0.01	9.60
Equatorial Guinea	0.16	0.00	0.09	0.28
Eritrea	0.09	–0.03	–0.03	0.31
Eswatini (Swaziland)	2.79	0.05	0.07	4.07
Ethiopia	0.60	–0.06	0.01	106.34
Gabon	0.30	0.00	–0.03	1.07
Gambia	0.93	–0.33	–0.33	3.50
Ghana	0.30	–0.04	–0.08	7.85
Guinea	0.29	0.02	0.02	5.25
Guinea-Bissau	0.22	0.00	0.00	0.57
Kenya	0.26	–0.01	0.00	14.80
Lesotho	1.20	0.55	0.55	0.90
Liberia	0.05	0.02	0.02	0.09
Madagascar	0.19	–0.03	–0.01	6.80
Malawi	0.05	0.00	0.00	0.77
Mali	0.04	–0.01	0.01	1.49
Mauritania	0.49	0.08	0.09	1.28
Mauritius	0.05	–0.01	–0.01	0.07
Mozambique	0.50	0.07	0.02	8.64
Namibia	5.57	–0.20	–0.05	17.57
Niger	0.00	0.00	0.00	0.03
Nigeria	0.07	–0.01	–0.01	17.19
Rwanda	0.21	–0.01	0.02	4.22
Sao Tome and Principe	0.60	0.07	0.07	0.03
Senegal	0.43	0.17	0.17	5.53
Seychelles	0.44	0.00	0.00	0.01
Sierra Leone	0.11	0.01	0.01	0.51
Somalia	0.02	–0.01	–0.01	0.87
South Africa	2.70	–0.07	–0.10	173.18
South Sudan	0.05	0.00	–0.01	0.36
Sudan	0.03	0.00	–0.01	3.53
Tanzania	—^a^	—	—	—
Togo	0.14	–0.01	–0.01	1.38
Uganda	0.39	0.01	–0.02	12.27
Zambia	0.69	–0.01	–0.11	8.12
Zimbabwe	0.18	–0.04	0.40	11.79

^a^Tanzania does not report COVID-19 cases.

**Table 4:** 7-day persistence difference.

Country	7-day persistence September 8, 2020, n	7-day persistence September 15, 2020, n
Nigera	20.5709689	17.18749
Kenya	21.4950277	14.79916
Namibia	21.7793534	17.57133
Ethiopia	136.703827	106.3378
South Africa	216.684664	173.1828

**Table 5:** Observed new daily and cumulative COVID-19 infections.

Country		COVID-19 infections September 8, 2020, n		COVID-19 infections September 15, 2020, n	
**New daily infections**					
	Kenya	151		—^a^	
	Cameroon	244		—	
	Nigeria	296		—	
	South Africa	1079		772	
	Ethiopia	1136		700	
	Uganda	—		145	
	Senegal	—		223	
	Mozambique	—		231	
**Cumulative COVID-19 infections**					
	Kenya		35,356		36,301
	Ghana		45,012		45,655
	Nigeria		55,456		56,478
	Ethiopia		60,784		65,486
	South Africa		640,441		651,521

^a^Country was not in top 5 for this day.

**Figure 3:**
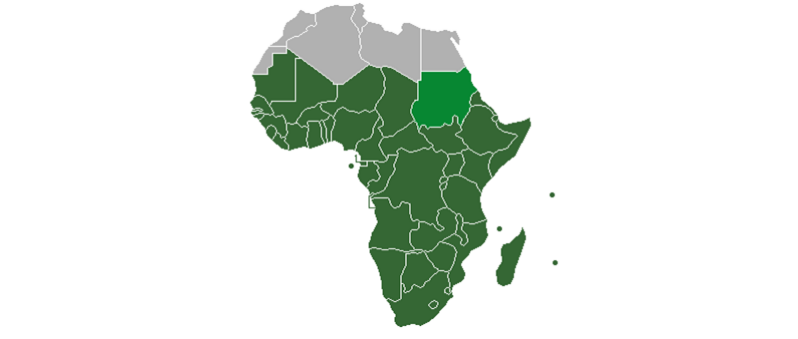
Sub-Saharan Africa region as defined by the United Nations.

The innovation of this study is the novel metrics we derived to measure how COVID-19 is spreading and those changes in the rates of transmission [75]. These measures should be considered in combination with traditional static numbers including observed new cases and deaths (see Figure 3). These novel metrics measure how fast the rates are changing [74].

We tracked the rates of COVID-19 transmission for SSA for the weeks of September 1-8 (see Table 2) and September 8-15, 2020 (see Table 3). The president of Tanzania has elected not to report COVID-19 morbidity and mortality, so this study is limited to the remaining 47 countries in SSA. SSA had 4206 new observed cases, with an SSA country average of 90 new cases the week of September 8, 2020, and 3156 new observed cases, with an SSA country average of 67 new observed cases the week of September 15, 2020, with significant variation between countries. The six countries with the highest rates of new infections per 100,000 population the week of September 8, 2020, includes Cape Verde, Namibia, Gambia, South Africa, Eswatini (formerly known in English as Swaziland), and Ethiopia at 7.6, 4.7, 3.3, 1.8, 1.7, and 1.0, respectively (see Table 2); whereas the following week (September 15, 2020), the six countries with the highest rates of new infections per 100,000 population were Cape Verde, Lesotho, Namibia, Eswatini, Senegal, and South Africa at 11.8, 3.9, 3.3, 2.1, 1.4, and 1.3, respectively. Gambia and Ethiopia dropped off the list of the top six countries with highest rates of new infections per 100,000 population, whereas Senegal and Lesotho were added within a 7-day span. Although the number of infections can be a function of time or a superspreader event that results in a one-time increase in COVID-19 infections, it is critical that we measure which country’s speed of COVID-19 transmission is *accelerating and decelerating* to inform public health policy. The six countries with the largest acceleration in rates of COVID-19 transmission are Sao Tome, Mauritania, Comoros, Senegal, Lesotho, and Cape Verde. In Table 3, we see that these six countries are accelerating at a rate of 0.066428, 0.075758, 0.100735, 0.169188, 0.551191, and 0.597473 per 100,000 population, respectively (see Table 2). To further identify countries that require immediate COVID-19 transmission mitigation and prevention strategies, we examined the *jerk.* The *jerk* measures the *change in* *acceleration or deceleration* week over week as the acceleration in the current week minus the acceleration of the previous week. The six highest jerks in positive acceleration rates between the weeks of September 8, 2020, and September 15, 2020, are Equatorial Guinea, Senegal, Zimbabwe, Lesotho, Cape Verde, and Congo-Brazzaville with 0.094818, 0.170064, 0.400904, 0.55119, 0.649427, and 0.682357 per 100,000, respectively (see Table 3). In sum, Cape Verde and South Africa have the highest rate of COVID-19 infections per 100,000 or rate of speed of COVID-19 transmissions per 100,000. Moreover, Senegal, Lesotho, and Cape Verde are rapidly accelerating and have the highest jerk rates, meaning that the acceleration of COVID-19 transmissions has jerked the acceleration rate upwards this past week relative to the previous week.

Controlling for incomplete case ascertainment and data contamination, we derived the 7-day persistence surveillance metric (see Table 4) [75]. The 7-day persistence explains the number of current COVID-19 infections that are a result of the number of COVID-19 infections 7 days ago [75]. Table 4 demonstrates the dynamic nature of the COVID-19 transmissions. On September 8, 2020, the five countries with the highest 7-day persistences were Nigeria, Kenya, Namibia, Ethiopia, and South Africa. For example, on September 8, 2020, South Africa had 216.7 new COVID-19 infections as a result of the number of COVID-19 infections from September 1, 2020. The following week, on September 15, 2020, these same five countries had the highest 7-day persistence based on the underlying condition that resulted in new infections a week ago. Thus, South Africa had 173.1 new cases based on the 1079 cases last week, Ethiopia has 106.3 new COVID-19 infections as a result of the new infections that occurred on September 8, 2020, and so on. The leading five countries with the highest persistence rates have all declined in the current week relative to the previous week.

Rank ordering sub-Saharan African countries by the number of observed daily new infections during the week ending on September 8, 2020, from least to most revealed that Kenya, Cameroon, Nigeria, South Africa, and Ethiopia ranked the highest (see Table 5). Whereas a week later the number of new infections had decreased, and three out of the five countries reporting observed daily infections had displaced Kenya, Cameroon, and Nigeria with Uganda, Senegal, and Mozambique. Another standard static surveillance metric is to enumerate the total number of COVID-19 infections or cumulative infections. The leading five nations with the total highest number of reported cases were Kenya, Ghana, Nigeria, Ethiopia, and South Africa (see Table 5).

The most populous countries in SSA include Kenya, Tanzania, South Africa, the Congo, Ethiopia, and Nigeria (see Table 6). Countries with larger populations are at risk for having more COVID-19 infections by virtue of size. Obviously, Tanzania is not reporting any cases of COVID-19 and, thus, was not part of this study; however, with the exception of the Congo, the other most populous countries in SSA had the most cases of COVID-19 infections over time (see Tables 5 and 6).

For comprehensive surveillance of static or traditional surveillance metrics with novel surveillance metrics for SSA, see Multimedia Appendix 1.

**Table 6:** Most populous sub-Saharan African countries.

Country	Population as of 2020, n
Kenya	52,573,973
Tanzania	58,005,463
South Africa	58,558,270
Congo	86,790,567
Ethiopia	112,078,730
Nigeria	200,963,599

## Discussion

### Principal Findings

The COVID-19 pandemic poses a threat to public health, economies, and food security around the world. Unfortunately, COVID-19 has reversed some positive gains made in SSA over the past few years in reversing extreme poverty and reducing food insecurity. The best hope for preventing further COVID-19 transmissions and mitigating the current adverse outcomes is through good public health surveillance coupled with enforced policies. Standard surveillance metrics such as daily observed new COVID-19 cases or deaths are necessary but insufficient to mitigate and prevent COVID-19 transmission. Public health leaders also need to know where COVID-19 transmission rates are accelerating or decelerating, whether those rates increase or decrease over short time frames because the pandemic can quickly escalate, and how many current cases are a function of new infections from 7 days ago.

Existing surveillance is helpful as it provides a single lens of the pandemic. For example, Nigeria has the largest population in SSA; South Africa has less than one-third of Nigeria’s population (see Table 6), yet Nigeria only has 56,478 cases (~12%) of the number of observed COVID-19 cases that South Africa has reported (n=651,521). These numbers indicate there is some underlying process that has resulted in South Africa having a disproportionate number of COVID-19 cases relative to its population size.

What standard surveillance does not reveal is if the rate of COVID-19 transmission is accelerating or decelerating, or if the rate of acceleration is increasing (jerk), resulting in explosive growth in COVID-19 transmissions. Although the basic reproduction number is a metric of transmission, it is based on missing data, asymptomatic infections, and data contamination [74]. Our analysis of the 7-day persistence that measures the persistence of COVID-19 given the number of new COVID-19 infections a week ago, revealed that the number of COVID-19 infections was declining. Moreover, when we examined the acceleration rate of COVID-19 transmissions in South Africa, we found that the rate of transmission was decelerating. Moreover, during the week of September 15, 2020, there was further evidence that the rate had reversed because the jerk was now negative. The acceleration of the current week, which was negative, minus the rate of acceleration of the previous week was also negative and means the COVID-19 transmission was decelerating and that the rate had increased further. From a public health perspective, even though South Africa has the highest number of total COVID-19 infections since January 2020, the rates of transmission have reversed. There remains a significant and large population of infected persons in South Africa that could result in additional outbreaks without proper adherence to public health guidelines that prevent the spread of COVID-19, but if public health leaders and the public remain resolute with maintaining recommended guidelines and prevention efforts, the numbers of COVID-19 infections will continue to decline.

What standard public health surveillance indicators overlook are those countries who have lower numbers of infections and, thus, do not appear concerning. However, our measures of COVID-19 transmission indicate an alarming acceleration rate in some countries. The six countries with the largest COVID-19 transmission acceleration rates are Sao Tome, Mauritania, Comoros, Senegal, Lesotho, and Cape Verde. Half of these countries are islands, suggesting that initially these island nations were successful at locking down the borders, but now, people with COVID-19 have penetrated the natural barriers that come from being on an island and the novel coronavirus is spreading quickly. Mauritania and Senegal are located in West Africa and share a border, which again suggests that the virus transmission is accelerating after maintaining initial control.

The six highest jerks in positive acceleration rates between the weeks of September 8, 2020, and September 15, 2020, are Equatorial Guinea, Senegal, Zimbabwe, Lesotho, Cape Verde, and Congo-Brazzaville. These six countries lead SSA in increased rates of acceleration this week minus the acceleration from last week, which is indicative of a much quicker rate of transmission that results in explosive growth of COVID-19 transmission rates. From a policy perspective, these countries should increase and enforce strict prevention measures such as quarantine or stay at home, avoiding crowds or large gatherings, social distancing, hand hygiene, and wearing face masks to flatten the curve or reverse current transmission trends. In summary, the number of COVID-19 infections and deaths are important surveillance indicators; however, measuring acceleration and deceleration rates, the jerk in rates, and the 7-day persistence can inform public health policy to mitigate outbreaks and prevent further COVID-19 transmission. Our novel indicators provide additional lens to understand how the pandemic is spreading and to inform policy and actions.

Finally, although we have identified countries that would benefit from informed policies, it merits reckoning that even though SSA is home to some of the poorest countries in the world, development and population size are not necessarily related to the rate and extent of COVID-19 transmission. The youthful population, hot climate, and the number of Africans living and working in rural areas serves as potential protective factors. Of note, this study also confirms the power of public health surveillance and policy. Existing surveillance and diagnostic testing systems for diseases such as TB have pivoted toward COVID-19, giving SSA an advantage over countries without existing infectious contagious diseases. Countries such as the United States should study how several low-income countries with fewer resources are able to prevent and control the spread of COVID-19 in efficient and cost-effective ways.

### Limitations

Our data are available at the country level, meaning reported observed cases and rates of transmission are the country’s average. Additional data collection and analysis are necessary to understand variations of COVID-19 transmission within countries. In addition, due to limitations in some country’s health care infrastructure, several days of new COVID-19 infections and deaths may have been reported on a single day. To that end, we reported average daily cases to control for batch reports.

### Conclusion

Public health surveillance systems are necessary to guide our leaders during disease outbreaks. Unfortunately, traditional surveillance metrics have undercounts, errors, and other data contamination that this study overcomes by using dynamic panel data and generalized method of the moments. Our methods control for data limitations and incomplete case ascertainment that exist in traditional surveillance systems.

Standard surveillance system metrics such as the prevalence and cumulative numbers of COVID-19–related morbidity and mortality are helpful; however, public health surveillance systems are limited in that they pick up the most severe cases. Incomplete case ascertainment is likely more pronounced during the COVID-19 pandemic where significant portions of the world’s population infected with the novel coronavirus remain asymptomatic or presymptomatic. Even in a perfect world where surveillance metrics can enumerate all novel cases and deaths, important information is needed to guide mitigation and prevention policies. This study addresses that dearth by applying novel surveillance indicators including speed, acceleration or deceleration, jerk, and persistence of cases at a 7-day persistence time that measures the number of current new cases as a function of the number of new cases 7 days ago. Knowing the number of new cases or the total number of COVID-19 cases can be misleading. It is possible to have fewer total cases and be at risk for exponential growth and greater population health impact if the speed of the COVID-19 transmission is positive and if the speed of the COVID-19 transmission is accelerating, and moreover, if the speed is accelerating and that rate has positive jerks after subtracting out the acceleration rate from the prior week. It is critical that we understand how COVID-19 is spreading, and our study provides useful metrics to accomplish better surveillance in SSA.

## Appendix

Multimedia Appendix 1 Sub-Saharan Africa COVID-19 trends.

## Abbreviations

Africa CDC: Africa Centres for Disease Control and Prevention
SSA: sub-Saharan Africa
TB: tuberculosis
WHO: World Health Organization
