# Supplementary figures and images for: A SARS-CoV-2 Surveillance System in Sub-Saharan Africa: Modeling Study for Persistence and Transmission to Inform Policy
Source: J Med Internet Res. 2020 Nov 19;22(11):e24248. doi: 10.2196/24248 (PMC7683024; doi:10.2196/24248)

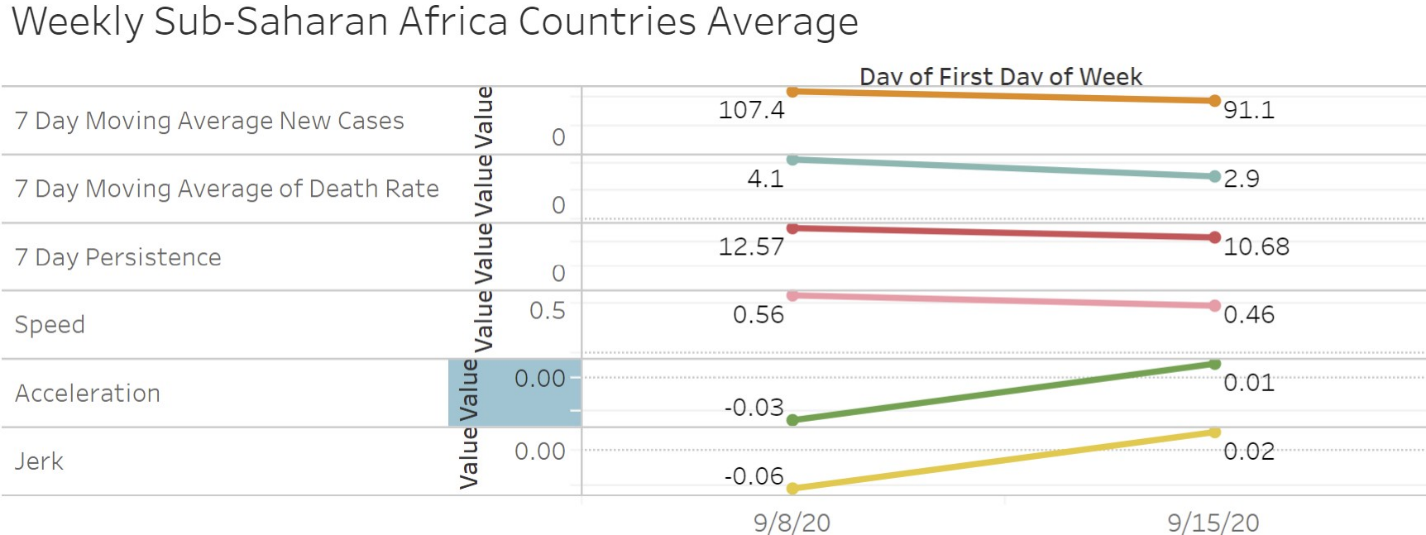


Weekly Country Trends


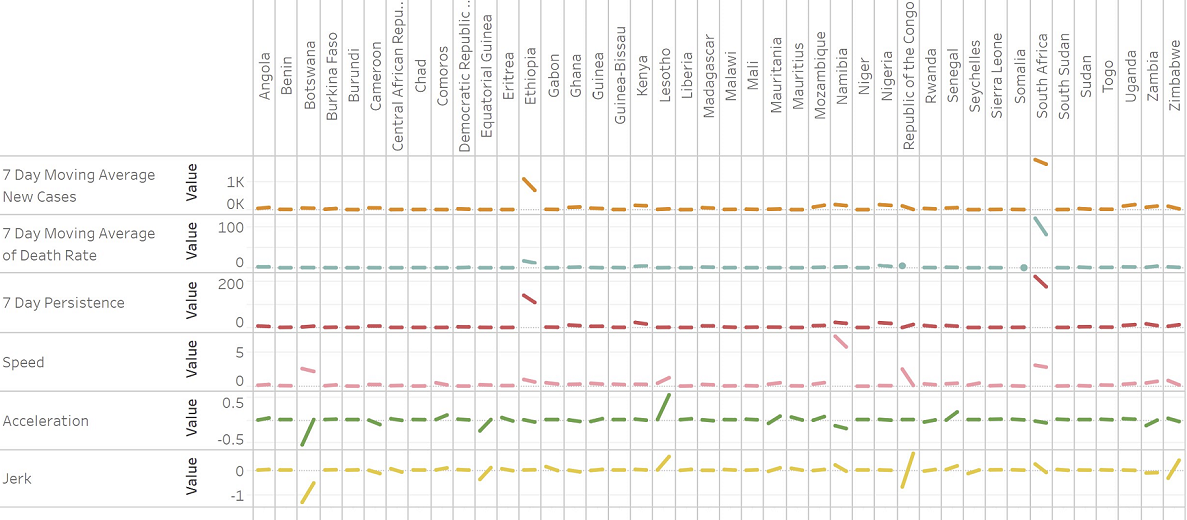


Weekly Africa Maps-9-08-2020


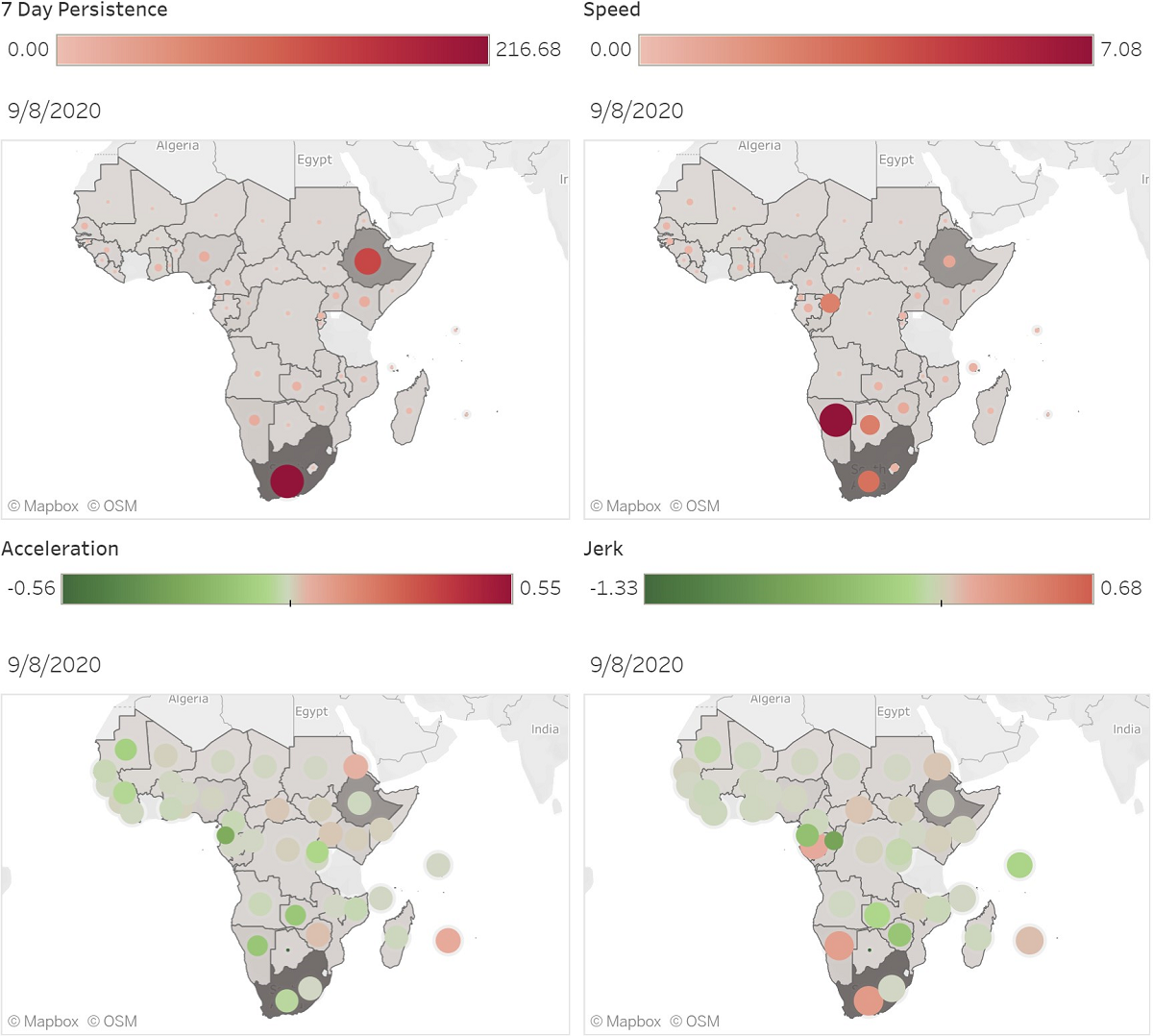


Weekly Africa Maps-9-15-2020


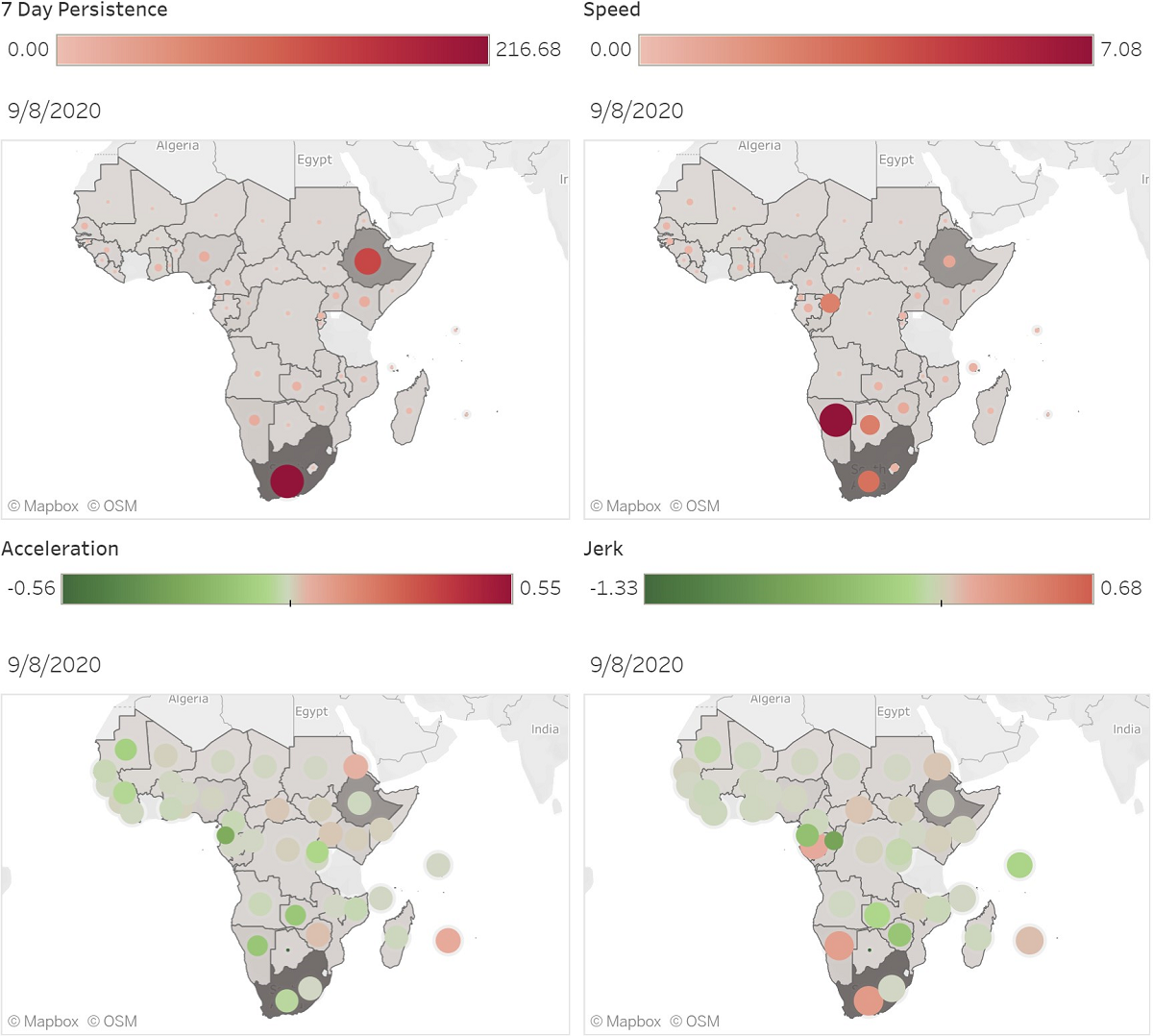


Daily Trends


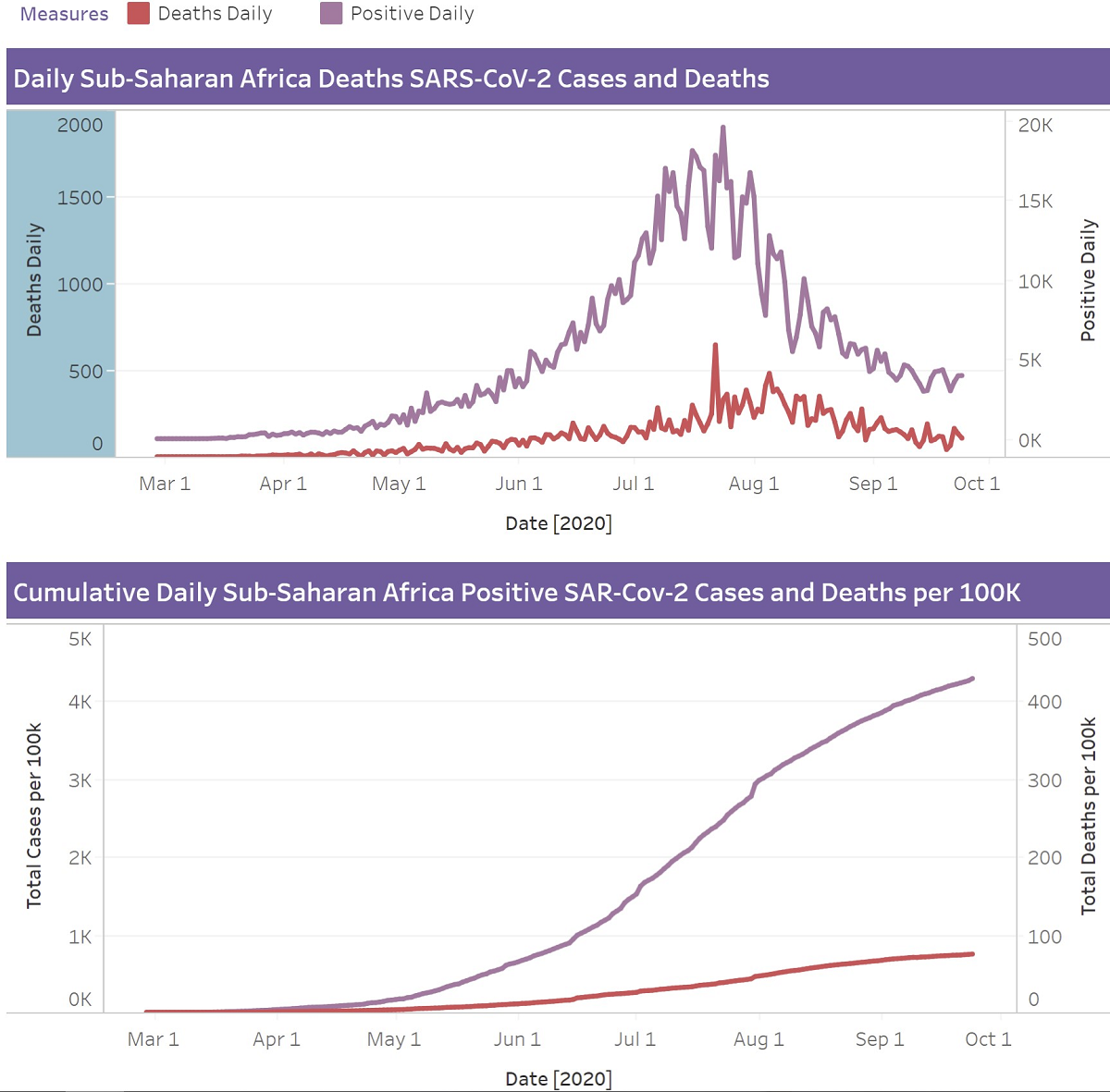


Daily Maps


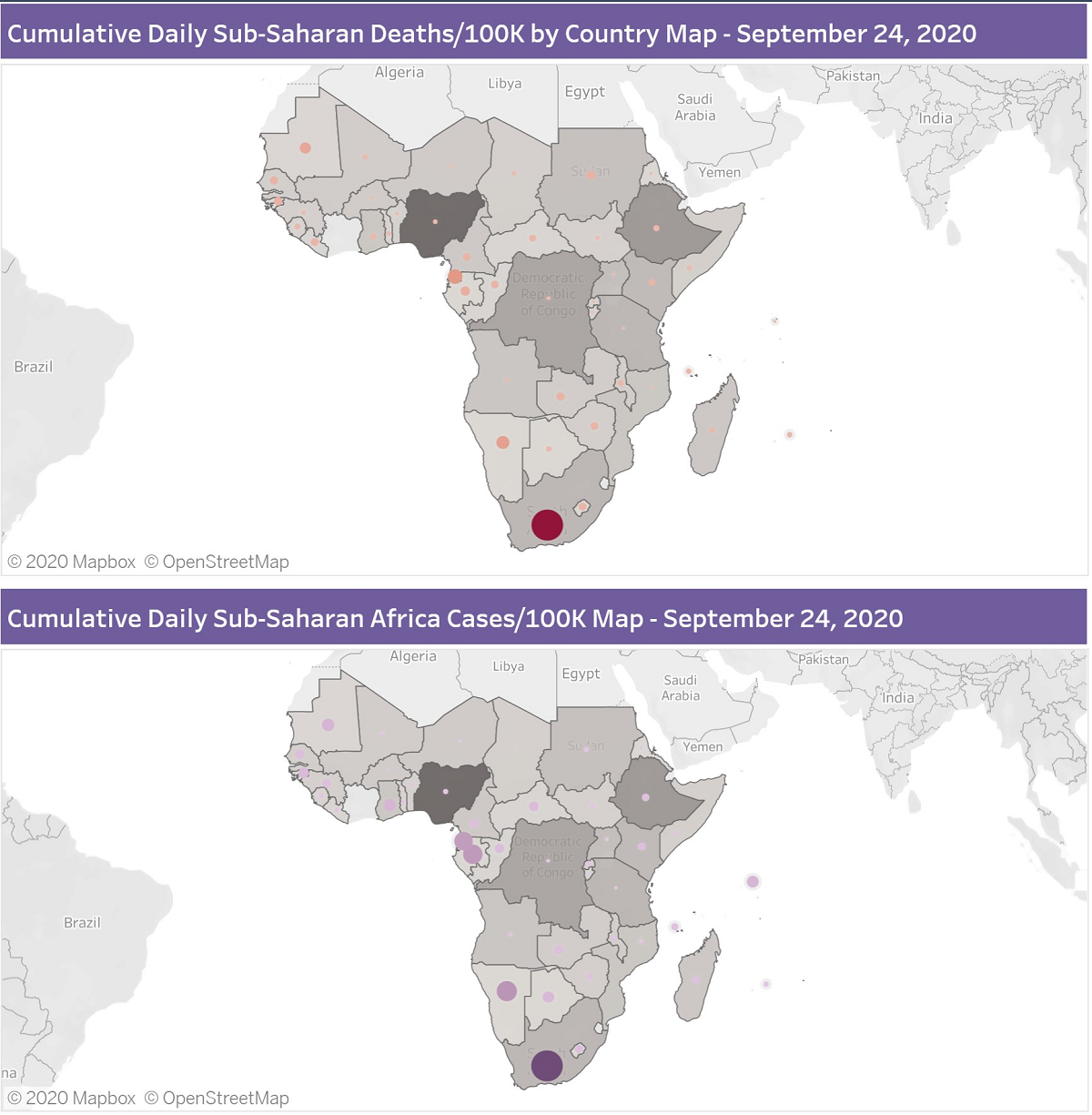

Supplement: Multimedia Appendix 1 [file jmir_v22i11e24248_app1.docx]
